# HACCP-compatible ear acupuncture using biodegradable starch-based needles mitigates transport-induced physiological and oxidative stress in calves

**DOI:** 10.14202/vetworld.2026.380-388

**Published:** 2026-01-30

**Authors:** Shogo Sato, Chihiro Kanno, Yurika Hoshi, Osamu Yamato, Moe Ijiri, Hiroshi Miura, Hisaya K Ono, Fumiaki Takahashi, Hiroaki Kawaguchi

**Affiliations:** 1 Laboratory of Clinical Veterinary Medicine for Large Animals, Kitasato University School of Veterinary Medicine, Aomori, Japan; 2 Laboratory of Veterinary Pathology, Kitasato University School of Veterinary Medicine, Aomori, Japan; 3 Laboratory of Clinical Pathology, Joint Faculty of Veterinary Medicine, Kagoshima University, Kagoshima, Japan; 4 Transboundary Animal Diseases Research Center, Joint Faculty of Veterinary Medicine, Kagoshima University, Kagoshima, Japan; 5 Laboratory of Theriogenology, Kitasato University School of Veterinary Medicine, Aomori, Japan; 6 Laboratory of Zoonoses, Kitasato University School of Veterinary Medicine, Aomori, Japan

**Keywords:** animal welfare, antioxidant capacity, biodegradable needles, cattle transport, ear acupuncture, HACCP-compliant technology, livestock stress management, oxidative stress biomarkers, oxytocin response, physiological stress, starch-based needles, transport stress in calves

## Abstract

**Background and Aim:**

Transportation is an unavoidable management practice in cattle production and is a major source of physiological, endocrine, and oxidative stress, leading to impaired welfare, immunity, and productivity. Drug-free and food-safety-compatible interventions to alleviate transport stress remain limited. Acupuncture has shown stress-reducing effects in livestock; however, conventional metal needles are incompatible with Hazard Analysis and Critical Control Point (HACCP) systems. Recently developed biodegradable starch-based Circular transdermal needles (CTNs) offer a novel HACCP-compliant alternative. This study aimed to evaluate the effects of ear acupuncture using starch-based CTNs on transport-induced stress responses and oxidative status in calves.

**Materials and Methods:**

Five clinically healthy male Holstein calves (3–4 months old) were subjected to a crossover experimental design involving a short-distance transport challenge (2 h 20 min). Calves received either ear acupuncture at the Jisen acupoints using starch-based CTNs or no treatment (control), with a washout period between treatments. Heart rate and rectal temperature were recorded, and blood samples were collected prior to transport (PRE), immediately after transport (POST1), and 2 days after transport (POST2). Serum stress markers (cortisol, catecholamines, oxytocin), oxidative stress indices (derivatives of reactive oxygen metabolites [d-ROMs], biological antioxidant potential [BAP], and BAP/d-ROMs ratio), and biochemical parameters were analyzed. Data were expressed as relative changes between time points and statistically evaluated at p < 0.05.

**Results:**

Acupuncture treatment suppressed post-transport increases in heart rate and rectal temperature compared with controls. Cortisol responses were attenuated in the acupuncture group, while oxytocin levels were consistently higher, indicating enhanced stress tolerance. Importantly, the BAP/d-ROMs ratio was significantly higher in acupuncture-treated calves at POST1 and POST2, reflecting improved oxidative balance and reduced oxidative stress. Although serum amyloid A increased slightly after acupuncture, no local inflammation or adverse reactions were observed at needle insertion sites.

**Conclusion:**

Ear acupuncture using biodegradable starch-based CTNs effectively mitigated physiological, endocrine, and oxidative stress responses associated with short-distance transport in calves. This HACCP-compatible, drug-free approach represents a novel and practical strategy to enhance animal welfare and stress resilience in cattle production systems.

## INTRODUCTION

Livestock animals, including cattle, pigs, and horses, are increasingly subjected to transportation as part of modern production and management systems. Numerous studies have demonstrated that transportation induces physiological stress, as evidenced by elevated blood cortisol concentrations across multiple animal species, including cattle, confirming transport as a significant stressor [1–7]. Addressing transport-associated stress is therefore essential to improve animal welfare and maintain productivity in livestock systems.

We previously developed an acupuncture-based intervention to reduce transport stress in pigs [3, 4]. This technique involves the insertion of Circular transdermal needles (CTNs) into the apical region of both ears at specific acupuncture sites corresponding to Erjian points one to three, referred to as “Jisen” in Japanese [3, 4, 7]. Stimulation of the Jisen acupoints influences the hypothalamic center and vagus nerve and is associated with somatic autonomic reflexes, including the modulation of gastric acid secretion. Experimental evidence from microminipigs demonstrated that this acupuncture treatment suppresses hypothalamic–pituitary–adrenal (HPA) axis activity and alleviates transport-induced stress without altering central catecholaminergic function [3]. Acupuncture is particularly advantageous in livestock production because it avoids drug residues and adverse pharmacological effects.

However, the use of conventional stainless-steel needles pose challenges for compliance with the Hazard Analysis and Critical Control Point (HACCP) management system. To overcome this limitation, biodegradable starch-based CTNs were developed. Unlike traditional stainless steel or polymer devices, starch-based CTNs are fully biodegradable and HACCP-compliant, eliminating concerns related to needle residue and foreign body contamination. This innovation makes acupuncture more feasible for use in food-producing animals under stringent safety regulations.

Oxidative stress plays a critical role in the pathogenesis of numerous human diseases [8]; however, its relationship with transport stress in livestock remains poorly understood. The biological antioxidant potential (BAP)/derivatives of reactive oxygen metabolites (d-ROMs) ratio is a valuable indicator of systemic oxidative balance, yet it has been investigated in only a limited number of cattle studies [9–11]. Importantly, transport-induced stress has been shown to impair cattle productivity and immune function [12], highlighting the need for further investigation into oxidative stress modulation during transportation.

Despite extensive evidence demonstrating that transportation induces physiological and endocrine stress responses in livestock, effective, practical, and food-safety-compliant mitigation strategies remain limited. Most previous studies on transport stress in cattle have focused primarily on classical stress indicators, such as cortisol and catecholamines, with comparatively little attention given to oxidative stress dynamics, despite their recognized role in immune suppression and productivity loss. In particular, the BAP/d-ROMs ratio has rarely been evaluated in the context of transport stress in cattle, leaving a substantial gap in understanding the redox imbalance associated with transportation.

Although acupuncture has emerged as a promising non-pharmacological approach to stress reduction in animals, existing studies have been largely restricted to laboratory species or pigs, and the translation of this technique to cattle remains poorly explored. Furthermore, conventional acupuncture devices, including stainless steel needles, pose challenges for implementation in food-producing animals due to their incompatibility with HACCP requirements and concerns about foreign body residues. The recent development of biodegradable starch-based CTNs offers a novel solution to these limitations; however, their efficacy in mitigating transport-induced physiological, endocrine, and oxidative stress responses in cattle has not been previously investigated. Collectively, the lack of integrated evaluations combining stress hormones, autonomic indicators, and oxidative stress biomarkers, together with the absence of HACCP-compatible acupuncture interventions in calves, represents a critical knowledge gap in livestock stress management research.

The present study was designed to address these gaps by evaluating the effects of ear acupuncture using biodegradable, HACCP-compatible starch-based CTNs on transport-induced stress responses in calves. Specifically, this study aimed to (i) assess the influence of starch-CTN acupuncture on physiological indicators of transport stress, including heart rate and rectal temperature; (ii) examine its effects on endocrine stress markers, namely cortisol, catecholamines, and oxytocin; and (iii) investigate changes in systemic oxidative status using d-ROMs, BAP, and the BAP/d-ROMs ratio following short-distance transportation. By integrating classical stress markers with oxidative stress indices, this pilot study sought to provide a comprehensive assessment of the stress-modulating potential of acupuncture in calves. The findings are expected to provide foundational evidence for a drug-free, welfare-oriented, and food-safety-compliant intervention to reduce transport stress in cattle.

## MATERIALS AND METHODS

### Ethical approval

This study was conducted in accordance with the Institutional Guidelines for Animal Experiments and complied with the Japanese Act on Welfare and Management of Animals (Act No. 105 and Notification No. 6). Ethical approval was obtained from the Animal Research Ethics Committees of the Kitasato University School of Veterinary Medicine (permit numbers: 21-077 [2022.2.3] and 22-005 [2022.6.2]). All procedures were performed in line with the Animal Research: Reporting of *In Vivo* Experiments 2.0 guidelines. The experiment used the minimum number of animals required, and calves were monitored throughout the study period for adverse clinical signs; animals were clinically assessed during housing and at each sampling time point, and no severe clinical abnormalities were observed.

### Study period and location

The study was conducted in March and April 2023 at the Kitasato University School of Veterinary Medicine, Aomori, Japan.

### Animals and housing conditions

Five male Holstein calves aged 3–4 months, with body weights ranging from 66.8 to 128.0 kg, were included in the study. All animals were clinically healthy and had not received vaccination or deworming treatments prior to the experiment. To ensure animal welfare, the study was conducted using the minimum number of animals required.

The calves were housed in groups of two to three in semi-open barns measuring 3.6–4.0 × 3.6 m. They were maintained in free pens, fed grass twice daily, and provided with tap water ad libitum throughout the study period.

### Experimental design

Prior to the efficacy trial, the safety of acupuncture treatment using newly developed starch-based CTNs was evaluated in cattle other than those used in the present study. No adverse effects, including hemorrhage or inflammation at the needle insertion sites, were observed during a 10-week observation period, indicating that the starch-CTNs were biodegradable and HACCP-compatible (data not shown).

**Figure 1:**
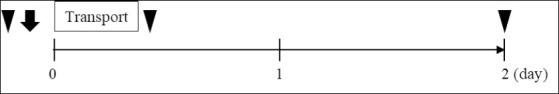
Experimental design. Animals were divided into a control group without treatment and an acupuncture group. Acupuncture was administered 30–60 min before transportation (arrow). Measurements of heart rate, rectal temperature, and blood collection were performed at three points (arrowheads).

For the efficacy evaluation (Figure 1), a crossover experimental design was employed. The five calves were assigned to either a control group without treatment or an acupuncture group receiving starch-CTN treatment. Initially, animals No. 1 and 2 received acupuncture, whereas animals No. 3–5 served as controls. After a washout period of more than one week, the treatments were reversed.

Heart rate, rectal temperature, and blood samples were collected at three time points: before acupuncture treatment and within 1 h prior to transport (PRE), immediately after transportation (POST1), and 2 days after transportation (POST2). Blood samples were collected from the jugular vein into plain tubes or ethylenediaminetetraacetic acid tubes (for catecholamine analysis) (Becton, Dickinson and Company, Franklin Lakes, NJ, USA) centrifuged at 678 × *g* for 15 min, and the resulting serum or plasma was stored at −30°C until analysis.

### Acupuncture procedure

**Figure 2:**
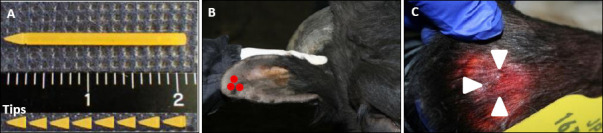
Acupuncture treatment. (A) New starch-circular transdermal needles. (B) Three red points indicate the ear acupoints Eisen). (C) The three tips of the needles are broken and placed in the subcutaneous tissue (arrowhead) while the light is shining from behind the ear.

Three starch-based CTNs (diameter × length: 1.5 mm × 20 mm) were applied to the apical region of each ear at locations corresponding to the Jisen acupoints 30–60 min before transportation (Figure 2), as previously described [3, 4, 7]. The needle tips (2 mm in length) were subcutaneously implanted during transport and removed 2 days after transportation.

### Transportation protocol

Calves were transported using a livestock transport vehicle (Mitsubishi Fuso Truck and Bus Corp., Kawasaki, Kanagawa, Japan; model PDG-FG84D) for approximately 2 h and 20 min on public roads, returning to the same housing facility. All transport operations were conducted in spring by the same experienced driver along an identical route to minimize variability related to driving style and road conditions [13].

The cargo bed measured 4.34 m in length, 1.85 m in width, and 2.11 m in height, providing a usable floor area of 8.03 m². The floor consisted of aluminum deck plates without bedding material. Calves were transported in groups of two or three, standing on the truck bed covered with a canvas tarpaulin and secured with ropes attached to nose rings.

### Physiological and biochemical measurements

Heart rate was measured by auscultation of heart sounds, and rectal temperature was recorded using a mercury thermometer. Both measurements were performed consecutively at each sampling time point.

Blood analyses included measurements of stress markers (cortisol, catecholamines, and oxytocin), biochemical parameters (serum amyloid A, total protein, and albumin), and oxidative stress indices (d-ROMs and BAP). The albumin/globulin (A/G) ratio and the BAP/d-ROMs ratio were calculated as previously described [3, 4, 14]. Outcome assessments were conducted with blinding to treatment allocation.

Changes in measured parameters were expressed as percentage increases or decreases relative to PRE for POST1/PRE, POST2/PRE, and POST2/POST1. Cortisol concentrations were determined by competitive enzyme-linked immunosorbent assay (ELISA) [15], and oxytocin levels were measured using a commercial ELISA kit (Enzo Biochem Inc., NY, USA). Serum amyloid A was quantified using an automated biochemical analyzer (Pentra C200; HORIBA ABX SAS, Montpellier, France) with a latex turbidimetric immunoassay reagent (VET-SAA “Eiken”; Eiken Chemical Co. Ltd., Tokyo, Japan) [3]. Oxidative stress parameters (d-ROMs and BAP) were assessed using a FREE CARRIO DUO Redox Analyzer system (Wismerll, Tokyo, Japan) according to the manufacturer’s instructions [3, 4]. Additional biochemical analyses were performed by a commercial laboratory (Hoken Kagaku, Inc., Kanagawa, Japan).

### Statistical analysis

All data are presented as the mean ± standard deviation. Homogeneity of variance was evaluated using the F-test. Differences between groups were analyzed using Student’s t-test for equivalent variances or Welch’s t-test for non-equivalent variances (JMP Pro 17.0.0; SAS Institute Inc., Cary, NC, USA). Statistical significance was set at p < 0.05, and values of 0.05 ≤ p < 0.1 were considered borderline significant. Effect sizes were calculated using Cohen’s d to quantify the magnitude of differences between groups (Table 1).

**Table 1:** Effect size and 95% confidence intervals of the ratio of heart rate, rectal temperature, and blood examination parameters between time points (Control vs Acupuncture group).

Parameter	POST1/PRE	POST2/PRE	POST2/POST1
Heart rate (bpm)	2.44 (117.5–145.4 vs 84.5–112.4)	1.06 (58.3–92.9 vs 76.0–110.7)	0.39 (82.3–112.2 vs 76.6–106.5)
Rectal temperature (°C)	0.82 (100.4–102.2 vs 99.8–101.5)	0.93 (98.6–100.8 vs 97.6–99.8)	1.42 (99.8–102.2 vs 98.1–100.5)
Cortisol (µg/dL)	0.31 (139.2–819.8 vs 36.6–717.1)	0.40 (9.0–64.9 vs 19.7–75.5)	0.11 (38.3–230.6 vs 48.6–240.9)
Adrenaline (ng/mL)	0.20 (23.99–259.4 vs 67.4–249.7)	0.21 (51.4–106.7 vs 56.9–112.2)	0.31 (50.5–210.6 vs 50.9–175.0)
Noradrenaline (ng/mL)	0.08 (89.0–260.7 vs 82.4–254.2)	0.41 (55.1–143.8 vs 37.4–126.2)	0.45 (29.9–372.5 vs −44.7–297.9)
Oxytocin (pg/mL)	0.49 (−32.1–192.9 vs 21.7–246.7)	0.66 (−184.3–370.4 vs −7.9–546.8)	0.49 (−905.7–1261.5 vs −386.1–1781.1)
Serum amyloid A (mg/L)	0.66 (−34.0–233.6 vs 51.1–318.7)	0.41 (41.1–276.2 vs −5.3–229.9)	0.435 (19.2–236.8 vs 65.1–282.7)
d-ROMs (U.CARR)	0.13 (95.4–105.9 vs 94.7–105.3)	1.02 (91.2–112.4 vs 80.1–101.9)	1.14 (92.3–111.7 vs 81.6–101.0)
BAP (µmol/L)	0.49 (85.4–100.3 vs 88.9–103.9)	0.80 (98.5–120.7 vs 89.9–112.1)	0.51 (93.4–108.8 vs 89.6–105.0)
BAP/d-ROMs ratio	0.78 (86.2–98.0 vs 90.7–102.5)	0.32 (95.3–120.3 vs 99.2–124.2)	0.59 (83.5–115.1 vs 92.6–124.2)

Effect size = Cohen’s *d*. Examinations were performed prior to transport (PRE), immediately after transportation (POST1), and 2 days after transportation (POST2). BAP = biological antioxidant potential, d-ROMs = derivatives of reactive oxygen metabolites.

## RESULTS

### Physiological responses to transport stress

**Figure 3:**
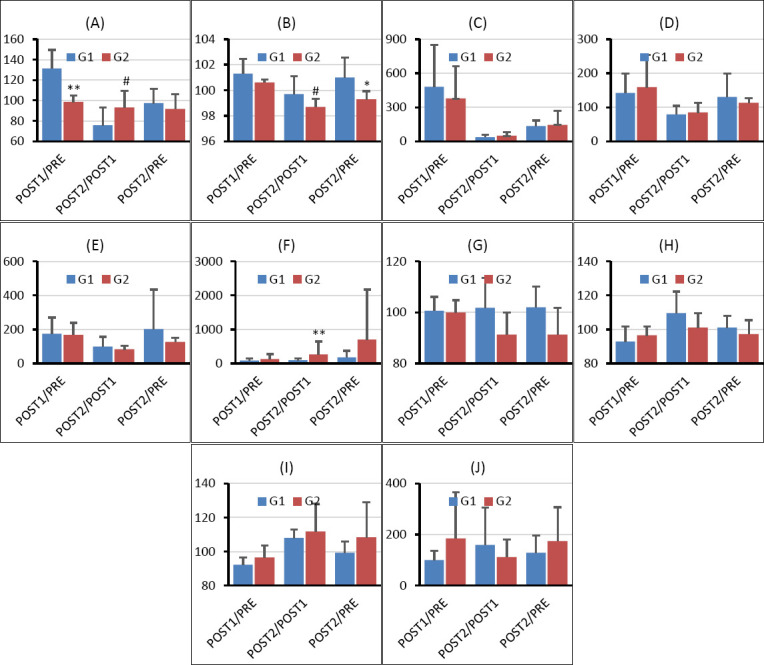
Effects of ear acupuncture using biodegradable starch-based circular transdermal needles on transport-induced physiological, endocrine, and oxidative stress responses in calves. **(A)** Heart rate, **(B)** rectal temperature, **(C)** serum cortisol concentration, **(D)** plasma adrenaline concentration, **(E)** plasma noradrenalin concentration, **(F)** serum oxytocin concentration, **(G)** derivatives of reactive oxygen metabolites (d-ROMs), **(H)** biological antioxidant potential (BAP), **(I)** BAP/d-ROMs ratio, and **(J)** serum amyloid A concentration are shown for the control group without treatment (G1, n = 5) and the acupuncture group (G2, n = 5). Measurements were obtained prior to transport (PRE), immediately after transportation (POST1), and 2 days after transportation (POST2). Data are presented as mean ± standard deviation. Statistical significance indicates comparisons between groups at the same time point: #0.05 ≤ p < 0.1, *p < 0.05, **p < 0.01.

Figure 3 illustrates the changes in heart rate, rectal temperature, and circulating stress markers, with corresponding mean values summarized in Table 2. The POST1/PRE and POST2/PRE ratios of heart rate were lower in the acupuncture group than in the control group, whereas the POST2/POST1 ratio was higher in the acupuncture group. In the control group, heart rate increased markedly following transportation, with a POST1/PRE ratio of 131%. In contrast, no post-transport increase was observed in the acupuncture group, where the POST1/PRE ratio remained at 98%. The POST2/POST1 and POST2/PRE heart rate ratios were below 100% in all calves, indicating recovery after transport.

**Table 2:** Average values ± standard deviation of heart rate, rectal temperature, and blood parameters.

Parameter	Control group PRE	Control group POST1	Control group POST2	Acupuncture group PRE	Acupuncture group POST1	Acupuncture group POST2
Heart rate (bpm)	71.2 ± 9.1	94.0 ± 21.0	68.4 ± 5.4	79.6 ± 13.2	78.0 ± 11.3	71.8 ± 9.1
Rectal temperature (°C)	38.7 ± 0.4	39.2 ± 0.4	39.1 ± 0.3	39.1 ± 0.3	39.4 ± 0.3	38.8 ± 0.2
Cortisol (µg/dL)	7.5 ± 3.7	27.9 ± 10.1	9.4 ± 4.5	11.5 ± 7.5	27.7 ± 9.6	11.8 ± 5.0
Adrenaline (ng/mL)	18.0 ± 8.7	22.2 ± 8.3	17.2 ± 7.5	15.2 ± 4.6	27.4 ± 26.8	17.2 ± 5.8
Noradrenalin (ng/mL)	63.6 ± 14.6	110.6 ± 65.1	133.2 ± 158.9	59.2 ± 8.0	102.2 ± 55.0	74.8 ± 17.5
Oxytocin (pg/mL)	0.16 ± 0.13	0.18 ± 0.15	0.16 ± 0.14	0.14 ± 0.10	0.12 ± 0.12	0.24 ± 0.42
Serum amyloid A (mg/L)	5.5 ± 5.3	5.4 ± 4.9	4.6 ± 1.8	7.9 ± 10.3	9.5 ± 12.7	7.5 ± 6.7
d-ROMs (U.CARR)	117.8 ± 14.6	118.6 ± 16.6	119.6 ± 11.5	125.0 ± 14.1	124.8 ± 14.0	113.4 ± 12.3
BAP (µmol/L)	2856.2 ± 211.9	2647.8 ± 261.2	2885.2 ± 260.3	2967.4 ± 218.3	2858.2 ± 226.5	2876.4 ± 181.6
BAP/d-ROMs ratio	24.5 ± 3.4	22.5 ± 2.9	24.2 ± 2.2	24.2 ± 5.0	23.2 ± 4.1	25.7 ± 3.6

Examinations were performed prior to transport (PRE), immediately after transportation (POST1), and 2 days after transportation (POST2). BAP = biological antioxidant potential, d-ROMs = derivatives of reactive oxygen metabolites.

### Rectal temperature and clinical observations

Throughout the experimental period, the mean rectal temperature of all calves was 39.1°C ± 0.4°C, and no instances of fever exceeding 40°C were recorded. Additionally, no diarrhea or other clinical abnormalities were observed. The POST1/PRE, POST2/POST1, and POST2/PRE ratios of rectal temperature were consistently lower in the acupuncture group compared with those in the control group, suggesting attenuation of transport-related thermoregulatory responses.

### Endocrine stress markers

Transportation induced pronounced increases in circulating stress hormones in the control group. The POST1/PRE ratios of cortisol, adrenaline, and noradrenalin were approximately 479%, 142%, and 175%, respectively. In the acupuncture group, the POST1/PRE ratio of cortisol was lower (377%) than that in the control group, indicating partial suppression of HPA axis activation. However, no significant differences were observed between groups in the POST1/PRE ratios of adrenaline or noradrenalin.

### Oxytocin response

The acupuncture group exhibited higher POST1/PRE, POST2/POST1, and POST2/PRE ratios of oxytocin compared with the control group. These findings indicate a sustained elevation of oxytocin following acupuncture treatment, consistent with enhanced stress-coping and calming responses.

### Oxidative stress indices

The POST1/PRE, POST2/POST1, and POST2/PRE ratios of the BAP/d-ROMs ratio were higher in the acupuncture group than in the control group, reflecting improved systemic redox balance. In addition, the POST2/POST1 and POST2/PRE ratios of d-ROMs were lower in the acupuncture group, indicating reduced oxidative stress during post-transport recovery.

### Inflammatory marker

The POST1/PRE and POST2/PRE ratios of serum amyloid A, an acute-phase inflammatory marker, were higher in the acupuncture group than in the control group, suggesting a mild acute response following acupuncture treatment.

## DISCUSSION

### Significance of transport stress mitigation in livestock

Mitigating transport-induced stress in livestock is essential to prevent deterioration in meat quality, reduce economic losses for producers, and improve overall animal welfare. This study provides the first evaluation of a drug-free acupuncture intervention using novel, biodegradable, and HACCP-compatible starch-based CTNs to reduce transport stress in cattle. Few stress-mitigation strategies evaluated in farm animals simultaneously satisfy biodegradability and HACCP requirements, positioning starch-CTNs as a unique and scalable welfare-oriented technology that does not compromise food chain safety. The present work represents a preliminary step in translating previously validated acupuncture-based stress reduction strategies from pigs [3, 4] to cattle.

### Effects on cardiovascular and thermoregulatory responses

The marked increase in the POST1/PRE heart rate ratio observed in the control group was consistent with transport-induced stress responses previously reported in cattle [16]. In contrast, acupuncture treatment effectively suppressed post-transport heart rate elevation, and this effect appeared to persist beyond transportation, as evidenced by POST2/POST1 and POST2/PRE ratios below 100% in all calves.

Although calves are known to develop fever and diarrhea following transportation [17], neither clinical sign was observed in this study. Nevertheless, rectal temperature ratios (POST1/PRE, POST2/POST1, and POST2/PRE) were consistently lower in the acupuncture group than in the control group, indicating that acupuncture attenuated transport-related thermoregulatory responses even in the absence of overt clinical illness.

### Modulation of endocrine stress markers

Transport stress was objectively confirmed by pronounced increases in circulating cortisol, adrenaline, and noradrenalin levels in the control group, with POST1/PRE ratios exceeding 100% for all three markers. Acupuncture treatment reduced the POST1/PRE cortisol response by approximately 21% compared with the control group, suggesting a modest but meaningful stress-relieving effect. This finding is consistent with previous reports indicating that acupuncture suppresses HPA axis activation without markedly altering catecholaminergic responses [3, 4].

### Oxytocin-mediated stress adaptation

Oxytocin responses were consistently higher in the acupuncture group across all evaluated ratios (POST1/PRE, POST2/POST1, and POST2/PRE), supporting a calming and stress-adaptive effect of acupuncture. Oxytocin, synthesized in the paraventricular and supraoptic nuclei of the hypothalamus, is known to downregulate HPA axis activity, modulate autonomic stress responses, attenuate inflammation, and reduce anxiety-related behaviors [18]. In dairy cattle, increased oxytocin secretion in response to psychological stressors has been associated with improved coping ability in novel environments [19]. The sustained elevation of oxytocin observed in acupuncture-treated calves suggests enhanced tolerance to transport-related environmental stress and implicates a neuroendocrine mechanism involving vagal and hypothalamic modulation that remains under-explored in livestock stress research.

### Influence on oxidative stress balance

A previous study in pigs demonstrated that transportation reduces the BAP/d-ROMs ratio, whereas acupuncture reverses this imbalance by improving oxidative status [3]. Similarly, in the present study, the reduction of the POST1/PRE BAP/d-ROMs ratio below 100% was attributed to transport stress, while acupuncture treatment mitigated this effect. Notably, the improvement in oxidative balance in calves was primarily associated with suppression of the decline in BAP, reflecting preserved antioxidant capacity. The combined assessment of classical stress markers (cortisol, catecholamines, and oxytocin) alongside oxidative stress indices (BAP/d-ROMs) in calves represents a novel and integrative approach rarely applied in transport stress research.

### Inflammatory response and safety considerations

The POST1/PRE and POST2/PRE ratios of serum amyloid A, an acute-phase inflammatory marker, were higher in the acupuncture group than in the control group. This increase was interpreted as a mild physiological response to the subcutaneous placement of starch-CTN tips rather than pathological inflammation, as no local inflammatory signs were observed at the acupuncture sites. Nonetheless, the interaction between acupuncture and immune responses warrants further investigation. No differences were detected in other biochemical parameters between groups, supporting the overall safety of the intervention. To our knowledge, this is the first study to evaluate starch-based CTN acupuncture as a stress-mitigation strategy in calves.

### Limitations and future perspectives

This study was conducted as a pilot investigation using a minimal sample size. Future research should include larger cohorts encompassing different breeds and age groups, extended transport durations, behavioral assessments, and evaluation under commercial transport conditions. Such studies will be essential to validate the reproducibility, scalability, and practical applicability of starch-CTN acupuncture in cattle production systems.

## CONCLUSION

This pilot study demonstrated that ear acupuncture using biodegradable, HACCP-compatible starch-based CTNs effectively mitigated transport-induced stress responses in calves. Acupuncture treatment suppressed post-transport increases in heart rate and rectal temperature and attenuated activation of the HPA axis, as evidenced by a 21% reduction in the cortisol POST1/PRE ratio compared with untreated controls. In addition, acupuncture-treated calves exhibited consistently elevated oxytocin responses across post-transport time points, suggesting enhanced stress tolerance and improved neuroendocrine adaptation. Importantly, systemic oxidative balance was improved in the acupuncture group, as reflected by higher BAP/d-ROMs ratios and lower post-transport d-ROMs levels during recovery. No adverse clinical effects or local inflammatory reactions were observed at the acupuncture sites, supporting the safety of the intervention.

The findings indicate that starch-CTN–based acupuncture represents a feasible, drug-free, and food-safety-compliant approach to reducing transport stress in cattle. Because the needles are biodegradable and HACCP-compatible, this method can be integrated into livestock production systems without concerns related to drug residues or foreign body contamination. The observed improvements in physiological stability, endocrine stress modulation, and oxidative balance suggest potential benefits for animal welfare, post-transport recovery, and maintenance of productivity, particularly during routine short-distance transportation.

A key strength of this study lies in its integrative assessment of transport stress, combining physiological indicators, classical endocrine markers, and oxidative stress indices within a single experimental framework. The use of a crossover design minimized individual variability, and the application of a novel biodegradable acupuncture device addressed a critical limitation associated with conventional acupuncture in food-producing animals. Furthermore, this study provides the first evidence supporting the efficacy of starch-based CTNs for stress reduction in calves.

In conclusion, ear acupuncture using starch-based CTNs offers a promising, welfare-oriented strategy for mitigating transport-induced stress and oxidative imbalance in calves. Although conducted as a pilot study with a limited sample size, these findings provide foundational evidence for the broader application of HACCP-compatible acupuncture in cattle. Further large-scale and field-based studies are warranted to confirm its effectiveness across diverse production settings and to support its incorporation into routine livestock transport management practices.

## DATA AVAILABILITY

The supplementary data can be made available from the corresponding author upon request.

## AUTHORS’ CONTRIBUTION

SS: Investigation, methodology, and drafted the manuscript. CK, MI, and YH: Investigation and drafted the manuscript. OY: Investigation, supervision, drafted and revised the manuscript. HM, FT, and HKO: Revised the manuscript. HK: Conceptualization, data curation, formal analysis, investigation, methodology, supervision, and drafted the manuscript. All authors have read and approved the final version of the manuscript.
